# Robot-Assisted Eye Surgery: A Systematic Review of Effectiveness, Safety, and Practicality in Clinical Settings

**DOI:** 10.1167/tvst.13.6.20

**Published:** 2024-06-25

**Authors:** Arun J. Thirunavukarasu, Monica L. Hu, William P. Foster, Kanmin Xue, Jasmina Cehajic-Kapetanovic, Robert E. MacLaren

**Affiliations:** 1 Nuffield Laboratory of Ophthalmology, Nuffield Department of Clinical Neurosciences, University of Oxford, Oxford, UK; 2 Oxford University Clinical Academic Graduate School, University of Oxford, Oxford, UK; 3 University of Cambridge School of Clinical Medicine, University of Cambridge, Cambridge, UK; 4 Department of Physiology, Development and Neuroscience, University of Cambridge, Cambridge, UK; 5 Oxford Eye Hospital, Oxford University Hospitals NHS Foundation Trust, Oxford, UK

**Keywords:** robotic systems, eye surgery, robotic surgery, robot-assisted surgery, ophthalmic surgery

## Abstract

**Purpose:**

Surgical innovation in ophthalmology is impeded by the physiological limits of human motion, and robotic assistance may facilitate an expansion of the surgical repertoire. We conducted a systematic review to identify ophthalmic procedures in which robotic systems have been trialled, evaluate their performance, and explore future directions for research and development of robotic techniques.

**Methods:**

The Cochrane Library, Embase, MEDLINE, Scopus, and Web of Science were searched. Screening adhered to five criteria: (1) English language; (2) primary research article; (3) human patients; (4) ophthalmological surgery; and (5) robot-assisted surgery. Quality assessment was conducted with Joanna Briggs Institute Tools for Critical Appraisal. The study protocol was registered prospectively (PROSPERO ID CRD42023449793).

**Results:**

Twelve studies were included. In comparative studies, there was no difference in the occurrence of ocular harms in robot-assisted procedures and conventional surgery. However, robotic assistance did not demonstrate consistent benefits over manual surgery in terms of effectiveness or practicality, likely reflecting the learning curve associated with these systems. Single studies indicated the potential of robotic assistance to improve the consistency of subretinal drug infusion and efficiency of instrument manipulation in vitreoretinal surgery.

**Conclusions:**

Proof-of-concept studies have demonstrated the potential of robotic assistance to facilitate procedures otherwise infeasible or impractical, and may broaden access to surgery. However, robot-assisted surgery has not yet demonstrated any significant benefits over standard surgical practice. Improving the speed and reducing perioperative requirements of robot-assisted surgery are particular priorities for research and innovation to improve the practicality of these novel techniques.

**Translational Relevance:**

This systematic review summarizes the potential and limitations of robotic systems for assisting eye surgery and outlines what is required for these systems to benefit patients and surgeons.

## Introduction

Robotic surgery is becoming increasingly common around the world, with over a million robot-assisted procedures performed per year.^1^ Various types of robotic surgical system for ophthalmology have been designed, ranging from handheld instruments with robotic stabilizing elements to telemanipulation systems with surgeons exerting control of surgical instruments via a detached console.^2^^,^^3^ Despite these developments, adoption of robotic systems for assistance with ocular surgery has been limited, perhaps due to specific challenges: a small and rotationally mobile surgical field, delicate internal structures that must be preserved, and procedures conducted as quickly as within 15 minutes with patients often awake throughout.^4^ Moreover, successful intraocular surgery has little tolerance for movement proximal to entry ports on the globe, unlike other minimally invasive procedures that have exhibited widespread adoption of robotic systems.^5^ However, physiological limitations of human surgeons are beginning to limit innovation and safety. Physiological tremor of expert surgeons has been shown to limit performance in routine surgery, and new procedures demanding even greater precision may not be possible until such limitations can be overcome.^6^ By mitigating these difficulties, robotic systems offer a potential strategy to extend the abilities of eye surgeons.

Surgical procedures in ophthalmology are diverse, and various proof-of-concept reports have demonstrated the feasibility of robot assistance in procedures involving the anterior and posterior segments of the eye, as well as the surrounding orbital adnexa.^7^^–^^10^ However, robotic assistance is not currently a feature of routine clinical practice in ophthalmology. It is unclear whether or not robotic interventions can improve clinical outcomes, logistical parameters such as procedure duration, or surgical ergonomics.

Here, a systematic review was undertaken of the evidence base for robotic surgical interventions in ophthalmology. Specifically, clinical studies were examined to identify procedures in which surgical assistance has been applied, explore the endpoints used to quantify potential benefits conferred by robotic systems, and analyze the benefit of robot-based interventions relative to conventional surgery in terms of effectiveness, practicality, and safety. The primary aims of the review were to establish whether any robot assistance for eye surgery is supported by high-quality evidence, appraise measured outcomes as an indication of expected or potential benefits, and explore what further research and development are required to produce useful robotic systems for ophthalmological surgeons.

## Materials and Methods

### Search and Screening

The systematic review protocol was published prospectively on PROSPERO (identifier CRD42023449793), and the study adhered to PRISMA guidance. The Cochrane Library, Embase, MEDLINE, Scopus, and Web of Science were searched on August 6, 2023, using the search strategy presented in Supplementary Material S1. The search strategy combined key words and Medical Subject Headings (MeSH) corresponding to three themes: ophthalmology and eye disease (including all subspecialties), surgical procedures, and robotic systems. Duplicates were removed by a single researcher using Zotero (version 6.0.27-beta.3+3e12f3f20; Digital Scholar, Vienna, VA). Two independent and blinded researchers conducted abstract screening in Rayyan and on an online spreadsheet, with disagreement resolved by a third independent researcher casting an arbitrating vote.^11^ Included articles fulfilled the following inclusion criteria, with no restrictions on participant characteristics:

1. Is a primary research article
2. Is written in the English language
3. Involves human patients
4. Involves ophthalmological surgery
5. Features robot-assisted surgery

### Data Extraction and Analysis

A single researcher undertook data extraction for each study, with a second independent researcher verifying all entries subsequently. The following data were collected: citation details, location of study, study design, participant characteristics (e.g., age, sex, indication for surgery), intervention details, comparators, outcome variables, outcome results, and free text describing positive or negative conclusions about the intervention. Clinical outcomes for which data were collected included surgical success rate, pre- and post-procedural visual acuity, duration of successful subretinal infusion, volume of successful subretinal infusion, number of retinotomies, change in central retinal thickness, decrease in retinal venous filling time (on fluoroangiography), and regression of exophthalmos, as well as the change in any patient-reported outcome measures (PROMs). The practicality outcomes were duration of surgery (and surgical steps) and surgeon-reported ease, practicality, and utility. Safety was assessed by collecting data for incidence of microtraumatic events during surgery, incidence of complications, and intraoperative blood loss. Ergonomics were assessed through measurement of the distance traveled by instruments during procedures and through scored interviews of surgeons. Researchers also undertook quality assessment of included studies using Joanna Briggs Institute Tools for Critical Appraisal (Supplementary Material S2).^12^^,^^13^

A narrative synthesis was planned due to anticipated heterogeneity in procedures, study designs, and outcomes. Studies were grouped by outcome variable type (clinical effectiveness, practicality, and safety), as well as by anatomical location and procedure details. Study design and quality assessment were considered in assessing the certainty of evidence, although a paucity of comparative studies merited consideration of all identified trials to appraise outcomes. Figures were created in R (R Foundation for Statistical Computing, Vienna, Austria) and with Affinity Designer 1.10.6 (Pantone LLC, Carlstadt, NJ).

## Results

### Literature Search and Study Selection

**Figure 1.**
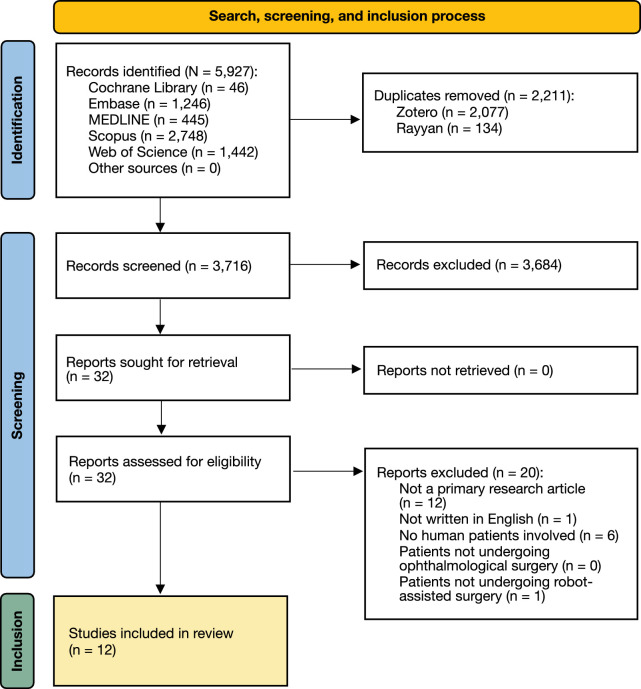
PRISMA flow chart depicting the search, screening, and inclusion process in this systematic review. Of 5927 records identified, 12 were included in the final synthesis: three RCTs, seven case series, and two case reports. The most common reasons for exclusion were lack of description of primary research and a lack of involvement of human patients.

The search and study selection process is illustrated in Figure 1. Of 3716 abstracts screened, reviewers exhibited disagreement in 20 cases, corresponding to a kappa statistic of 0.67 (substantial agreement). The most common reasons for exclusion of full texts were a lack of description of primary research data and a lack of involvement of human patients. Borderline cases of exclusion included robot-assisted radiotherapy (“radiosurgery”) and robotic surgery applied with recently deceased human patients or extracted human tissue.^14^^–^^16^ Twelve studies passed screening for final inclusion.^7^^–^^9^^,^^17^^–^^25^

### Study Characteristics and Quality Assessment

Characteristics of the included studies are summarized in Table 1. All but one study (from China) were conducted in Europe. Most studies were uncontrolled case reports or case series with sample sizes ranging between 1 and 10. There were three randomized controlled trials (RCTs), all utilizing the Preceyes Surgical System (Preceyes B.V., Eindhoven, Netherlands), with sample sizes between 12 and 15. The second most commonly applied robotic system was the da Vinci Surgical System (Intuitive Surgical, Sunnyvale, CA), which was applied in a variety of corneal and orbital procedures. Other systems included the KU Leuven robotic system (KU Leuven Research, Leuven, Belgium) and Medineering Robotic Endoscopy Guiding System (Medineering GmbH, Munich, Germany). Robotic assistance has been reported for a wide variety of ophthalmic procedures: orbital and lid surgery, ocular surface procedures, subretinal injection, and intravenous infusion, as well as epiretinal membrane (ERM) and internal limiting membrane (ILM) peeling. All three RCTs evaluated robotic interventions for vitreoretinal procedures: two for ERM or ILM peel and one for subretinal injection.^8^^,^^18^^,^^20^ Study design and quality of reporting ranged from fair to good, but limited sample size in all studies restricted the confidence of conclusions, particularly regarding clinical outcomes (Table 2). The most common reasons for poorer quality assessment scores were a lack of blinding of surgeons in RCTs; unclear selection protocols, demographic information, and diagnostic methods in case series; and lack of description of patients’ histories and adverse or unanticipated events in case reports.

**Table 1.** Characteristics of the 12 Studies Included in This Systematic Review

Citation	Design	Location	Intervention	Comparator	Diagnosis	Sample Size	Intervention: Control Ratio	Male: Female Ratio	Mean Age (y) (SD)	Valence of Discussion
Bourcier et al.^9^	Case report	Strasbourg, France	da Vinci Surgical System–assisted pterygium surgery	N/A	Pterygium	1	N/A	1:0	73	Neutral
Bourcier et al.^17^	Case series	Strasbourg, France	da Vinci Surgical System–assisted amniotic membrane transplant	N/A	Graft failure following penetrating keratoplasty for keratoconus; radiotherapy-induced keratoconjunctivitis sicca; herpetic keratitis	3	N/A	1:2	57 (22)	Neutral
Cehajic-Kapetanovic et al.^18^	RCT	Oxford, UK	Preceyes Surgical System–assisted subretinal injection of tissue-plasminogen activator	Conventional manual injection	Subfoveal hemorrhage secondary to neovascular age-related macular degeneration	12	1:1	Intervention, 4:2 Comparator, 3:3	Intervention, 75 (6.5) Comparator, 87.5 (4.9)	Positive
Cereda et al.^19^	Case series	Rotterdam, Netherlands	Preceyes Surgical System–assisted insertion of OCT sensor for intraoperative use	N/A	ERM; intraocular silicon oil; floaters	5	N/A	3:2	60.6 (8.4)	Positive
Edwards et al.^8^	RCT	Oxford, UK	Preceyes Surgical System–assisted ERM removal or internal limiting membrane peel	Conventional manual surgery	Macular hole requiring membrane peel for repair	12	1:1	Intervention, 4:2 Comparator, 1:5	Intervention, 62 (10) Comparator, 72 (8)	Neutral
Faridpooya et al.^20^	RCT	Rotterdam, Netherlands	Preceyes Surgical System–assisted ERM removal or internal limiting membrane peel	Conventional manual surgery	Idiopathic ERM	15	2:1	Intervention, 4:6 Comparator, 3:2	Intervention, 74 (3) Comparator, 73 (4)	Neutral
Gijbels et al.^21^	Case series	Leuven, Belgium	KU Leuven robotic system–assisted retinal venous cannulation and ocriplasmin infusion	N/A	Central retinal vein occlusion	4	N/A	Not reported	Not reported	Positive
Jeannon et al.^22^	Case report	London, UK	da Vinci Surgical System–assisted wide local excision of tumor	N/A	Basal cell carcinoma	1	N/A	0:1	85	Positive
Mattheis et al.^23^	Case series	Essen, Germany	Medineering Robotic Endoscope Guiding System–assisted orbital decompression	N/A	Graves’ orbitopathy	8	N/A	2:6	Not reported	Positive
Turgut et al.^24^	Case series	Zurich, Switzerland	Preceyes Surgical System–assisted ERM removal	N/A	Idiopathic ERM; macular hole; myopic macular schisis	9	N/A	Not reported	Not reported	Neutral
Wang et al.^7^	Case series	Shanghai, China	da Vinci Surgical System–assisted orbital fat decompression	N/A	Graves’ orbitopathy	10	N/A	0:10	30 (6.8)	Positive
Willekens et al.^25^	Case series	Leuven, Belgium	KU Leuven robotic system–assisted retinal venous cannulation and ocriplasmin infusion	N/A	Central retinal vein occlusion	4	N/A	3:1	70 (10)	Positive

Three randomized controls were identified, all involving application of the Preceyes Surgical System for vitreoretinal procedures. N/A, not applicable.

**Table 2.** Quality Assessment for All 12 Included Studies

(A) RCTs														
		Internal Validity												
		Selection and Allocation			Administration of Intervention			Assessment, Detection, and Measurement of Outcome			Participant Retention	Statistical Validity		
Citation	Outcome	1	2	3	4	5	6	7	8	9	10	11	12	13
Cehajic-Kapetanovic et al.^18^	Number of retinotomies	Yes	Yes	Yes	Unclear	No	Yes	Unclear	Yes	Unclear	Yes	No	Yes	Yes
	Volume of tPA injected	—	—	—	—	—	—	Unclear	Yes	Yes	Yes	No	Yes	—
	Duration of tPA injection	—	—	—	—	—	—	Unclear	Yes	Yes	Yes	No	Yes	—
	Duration of surgery	—	—	—	—	—	—	Unclear	Yes	Yes	Yes	No	Yes	—
	BCVA	—	—	—	—	—	—	Unclear	Yes	Yes	Yes	No	Yes	—
	Successful displacement of submacular hemorrhage	—	—	—	—	—	—	Unclear	Yes	Unclear	Yes	No	Yes	—
	Frequency of microtraumatic events	—	—	—	—	—	—	Yes	Yes	Yes	Yes	No	Yes	—
Edwards et al.^8^	Surgical success	Yes	Yes	Yes	Unclear	No	No	Unclear	Yes	Unclear	Yes	Yes	N/A	Yes
	Duration of surgery	—	—	—	—	—	—	No	Yes	Yes	Yes	Yes	Yes	—
	Time required to position instrument	—	—	—	—	—	—	No	Yes	Yes	Yes	Yes	Yes	—
	Time required to initiate membrane flap	—	—	—	—	—	—	No	Yes	Yes	Yes	Yes	Yes	—
	Frequency of microtraumatic events	—	—	—	—	—	—	No	Yes	Unclear	Yes	Yes	Yes	—
	Surgeons’ descriptive experiences	—	—	—	—	—	—	No	Yes	Unclear	Yes	Yes	N/A	—
Faridpooya et al.^20^	Feasibility	No	No	Yes	No	No	Yes	Unclear	Yes	Unclear	Yes	No	N/A	Yes
	Duration of surgery (and substeps)	—	—	—	—	—	—	Unclear	Yes	Yes	Yes	No	N/A	—
	Mean distance traveled by forceps	—	—	—	—	—	—	Unclear	Yes	Yes	Yes	No	N/A	—
	Change in BCVA	—	—	—	—	—	—	Unclear	Yes	No	Yes	No	N/A	—
	Change in central retinal thickness	—	—	—	—	—	—	Unclear	Yes	Unclear	Yes	No	N/A	—
	Frequency of adverse events	—	—	—	—	—	—	Unclear	Yes	Unclear	Yes	No	N/A	—
**(B) Case series**														
Citation	1	2	3	4	5	6	7	8	9	10				

Bourcier et al.^17^	No	Unclear	Unclear	Yes	Yes	Yes	Yes	Yes	No	Yes				
Cereda et al.^19^	No	No	Unclear	Unclear	Yes	Yes	Yes	Yes	No	Not applicable				
Gijbels et al.^21^	No	Unclear	Unclear	Unclear	Unclear	No	No	No	No	Not applicable				
Mattheis et al.^23^	No	Yes	Unclear	Unclear	Yes	Yes	Yes	Yes	No	Not applicable				
Turgut et al.^24^	No	Unclear	Unclear	Unclear	Yes	No	No	No	No	Not applicable				
Wang et al.^7^	Yes	Yes	Unclear	Unclear	Yes	Yes	Yes	Yes	No	Yes				
Willekens et al.^25^	Yes	Yes	Yes	Unclear	Yes	Yes	Yes	Yes	No	Yes				
**(C) Case reports**														
Citation	1	2	3	4	5	6	7	8						

Bourcier et al.^9^	No	No	Yes	Yes	Yes	No	No	Yes						
Jeannon et al.^22^	Yes	Unclear	Yes	Yes	Yes	Unclear	No	Yes						

Joanna Briggs Tools for Critical Appraisal were used for quality assessment; different instruments were employed for each study design observed. Quality of reporting ranged from fair to good, but sample size was ubiquitously low. BCVA, best-corrected visual acuity.

### Characteristics of Trialled Robotic Systems

Of the four trialled systems, the Preceyes Surgical System was featured most commonly.^8^^,^^18^^–^^20^^,^^24^ The Preceyes Surgical System makes use of a trocar holder and an integrated head rest to secure 23-gauge, 25-gauge, and 27-gauge instruments relative to the eye, permitting surgery under general or local anesthesia. Operators control instrument movement via a joystick and foot switch, and safety boundaries are encoded to prevent excessive instrument movement. The da Vinci Surgical System was the next most commonly featured, with the da Vinci Si patient cart in two studies for corneal surgery and da Vinci Xi patient cart in one study of orbital decompression surgery.^7^^,^^9^^,^^17^ The da Vinci Surgical System makes use of a separate surgeon console with two manual instrument manipulation handles and five pedals for other functions. Different patient carts can be linked to the console, with the Si and Xi systems offering three or four arms for instrumentation, respectively. Two consoles may be linked to allow surgeons to work together on a procedure with simultaneous visualization.^9^ The KU Leuven system, featured in two studies, features a joystick-less instrument which is both handled by the surgeon and stabilized by the robotic system. It facilitates locking of the instrument and eye to permit sustained tasks requiring high precision, such as retinal venous cannulation. The Medineering Robotic Endoscope Guiding System, since rebranded as Brainlab Cirq Robotics and marketed for spinal surgery, features a robotic arm affixed to the operating table which assists surgeons by providing a stable view of the surgical field, which is otherwise challenging with a manually controlled endoscope.

### Clinical Effectiveness

All three RCTs evaluated the efficacy of robot-assisted procedures relative to conventional surgery in curated settings rather than effectiveness in pragmatic settings. All three trials used the Preceyes Surgical System to perform either subretinal injection of tissue plasminogen activator (tPA) or ERM and ILM peel. Results concerning surgical success (e.g., tPA injection duration and volume), surrogate endpoints demonstrating effective treatment (e.g., central retinal thickness), and clinical endpoints (e.g., visual acuity) were similar between robotic and conventional surgical arms (Table 3). Specifically, outcomes following robot-assisted surgery were superior or equivalent to outcomes following manual surgery in six of seven comparisons; the lone exception was a marginal difference in the mean decrease in central retinal thickness after pucker peel surgery (99 µm vs. 125 µm for robot-assisted and conventional surgery, respectively).^20^ However, studies were not sufficiently powered to function as true superiority or non-inferiority trials.

**Table 3.** A Summary of Results From the 12 Included Studies

Domain	Study	Outcome	Robot-Assisted Arm	Control Arm	*P*
Clinical effectiveness	Edwards et al.^8^^*^	Surgical success	100%	100%	—
	Bourcier et al.^17^	Surgical success rate	100%	Not available	—
	Cehajic-Kapetanovic et al.^18^^*^	Median gain in visual acuity after 1 month	1.3 logMAR	1.62 logMAR	0.14
		Median duration of subretinal infusion	354 s	258 s	0.93
		Median volume of subretinal infusion	0.050 mL	0.100 mL	0.31
		Median number of retinotomies	1	2	0.34
	Willekens et al.^25^	Surgical success rate	100%	Not available	—
		Mean duration of subretinal infusion	355 s	—	—
		Mean decrease in central retinal thickness	584 µm	—	0.068
		Mean decrease in venous filling time on fluoroangiography	9 s	—	0.068
	Faridpooya et al.^20^^*^	Mean postoperative gain in visual acuity	4 lines	4 lines	—
		Mean postoperative decrease in central retinal thickness	99 µm	125 µm	—
	Cereda et al.^19^	Mean difference between robot-assisted and conventional measurement of retinal thickness	16.11 µm	Not available	—
	Wang et al.^7^	Mean regression of exophthalmos after 3 months	2.27 mm	Not available	0.0001
		Increase in mean GO-QoL visual function score	9.37	—	0.1875
		Increase in mean GO-QoL appearance score	21.88	—	0.027
Practicality	Edwards et al.^8^	Median total duration of surgery	55 minutes	31 minutes	<0.0001
		Median time for instrument to reach retina	146 s	12 s	0.002
		Median time to create elevated membrane flap	295 s	80 s	0.06
	Bourcier et al.^17^	Total duration of surgery	1827 s	Not available	—
	Cehajic-Kapetanovic et al.^18^	Mean total duration of surgery	42.7 minutes	46.9 minutes	0.61
	Faridpooya et al.^20^	Mean duration of preparation	63.9 minutes	12.9 minutes	—
		Mean time between instrument entry and exit	55.5 minutes	23.6 minutes	—
		Mean duration of ICG staining	42 s	31 s	—
		Mean time taken for ICG removal	94 s	48 s	—
		Mean time required for flap initiation	93 s	42 s	—
		Mean duration of ERM/ILM peeling	508 s	197 s	—
		Mean duration of illumination task	179 s	100 s	—
		Mean duration of fluid-air exchange	90 s	44 s	—
	Turgut et al.^24^	Mean time to prepare surgical system	12.3 minutes	Not available	—
		Mean time to prepare patient	47.2 minutes	—	—
		Mean duration of surgery	72.4 minutes	—	—
		Percentage agreement of surgeons that robot-assisted surgery was easier	11%	—	—
	Wang et al.^7^	Mean total duration of surgery	124.3 minutes	Not available	—
	Bourcier et al.^9^	Total duration of surgery	60.5 minutes	Not available	—
Safety	Edwards et al.^8^^*^	Median incidence of retinal microtrauma	0	1	0.2
	Cehajic-Kapetanovic et al.^18^^*^	Median incidence of retinal microtrauma	0	1	0.87
	Willekens et al.^25^	Incidence of complications	1 needle-tip breakage	Not available	—
	Wang et al.^7^	Mean intraoperative blood loss	17.8 mL	Not available	—
Ergonomics	Faridpooya et al.^20^^*^	Mean distance traveled by forceps	403 mm	550 mm	—
	Turgut et al.^24^	Percentage agreement of surgeons that robot-assisted surgery was less stressful	11%	—	—
		Percentage agreement of surgeons that robot-assisted surgery ameliorated hand and arm strain	89%	Not available	—
		Percentage agreement of surgeons that further robot-assisted surgery was desirable	78%	—	—

Three studies (all RCTs) provided a control arm for comparison of robot-assisted surgery to conventional procedures. In general, robotic systems were associated with similar effectiveness and safety as manual surgery but longer duration of procedures. ICG, indocyanine green.

* Randomised-control trials.

Four non-comparative studies assessed the clinical effectiveness of the Preceyes Surgical System and da Vinci Surgical System (Table 3). Where compared to preoperative assessment, surgery conferred measurable clinical benefit in every study, but statistically significant benefit was only observed in two of five comparisons: mean regression of exophthalmos and increase in GO-QoL appearance score after da Vinci Surgical System–assisted orbital fat decompression surgery.^7^ However, contextualization was limited by a lack of non-intervention comparators exposed to conventional management, no management, or placebo (sham management).

### Practicality

**Figure 2.**
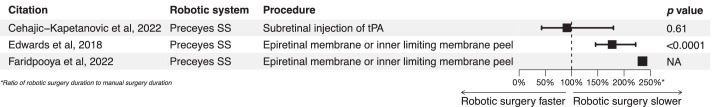
Forest plot comparing procedure duration of robot-assisted against conventional manual surgery. In two of three trials, robotic systems were associated with a significantly longer procedure duration, but in one trial robot-assisted surgery took less time than manual surgery (although this difference was not statistically significant). All trials concerned vitreoretinal procedures and featured the Preceyes Surgical System.

The most common measure of practicality was duration, either of entire surgical procedures or of components of procedures (Table 3). In 11 of 12 direct comparisons, all involving the Preceyes Surgical System, robot-assisted techniques required more time than manual surgery. In many cases, differences were statistically significant and often seemed impractical, with total duration of robot-assisted vitreoretinal surgery measuring up to 2.4 times as long as conventional surgery (Fig. 2).^8^^,^^20^ However, one study found that robot-assisted subretinal infusion of tPA required less time than conventional surgical techniques, although this difference was not statistically significant (Fig. 2).^18^ In addition to procedure duration, one study suggested that preparation time for robot-assisted ILM/ERM peel was five times longer than for manual surgery, which would represent a significant concern for teams aiming to incorporate a robotic system into their workflow.^20^

Anecdotes from case reports and case series testing the da Vinci Surgical System and Medineering Robotic Endoscopy Guiding System reiterated greater requirements for pre-procedural preparation and longer procedure duration but relative comfort and ease conferred by robotic systems.^7^^,^^9^^,^^23^ Investigators in trials of the da Vinci Surgical System and Preceyes Surgical System tended to agree that at least part of the longer duration of robot-assisted procedures was due to the learning curve associated with using a novel device, which would be expected to improve with experience.^9^^,^^20^ This is in contrast to the intensive training usually dedicated to perfecting manual surgical techniques undertaken over many years. In one of the RCTs trialling robotic assistance, procedure duration decreased remarkably with procedures conducted later, as surgeons developed familiarity and confidence using the novel system.^8^

Just one study compared the ergonomic value of robot-assisted surgery to conventional surgery. This study measured the total travel distance of instruments during surgery and found that procedures supported by the Preceyes Surgical System were more efficient in terms of distance of instrument movement (average = 403 mm) than conventional manual surgery (average = 550 mm) (Table 3).^20^ In another study, participating surgeons were surveyed about the use of a robotic system to assist vitreoretinal surgery,^24^ and 89% of surgeons agreed that robot-assisted surgery was associated with less physical strain and 78% agreed that more robotic assistance in ophthalmology was desirable. However, just 11% of surgeons agreed that robot-assisted surgery was less stressful than conventional surgery, although this may, again, be associated with a relative lack of experience with the robotic device.

### Safety

In general, very few complications were reported in the included studies. No complications with long-term effects on vision or wellbeing were recorded. Two studies evaluated the incidence of retinal microtrauma during vitreoretinal procedures, finding no difference between robot-assisted and manual surgery (Table 3).^8^^,^^18^ Needle-tip breakage was reported in one case report, but it was unclear whether this was attributable to robotic assistance, and no control arm was available to explore breakage rates with conventional surgery.^25^ In a case series (*n* = 10) of orbital surgery, intraoperative blood loss with robot-assisted surgery for Graves’ orbitopathy was measured, which was minimal in terms of mean (17.8 mL) and maximum (28 mL) (Table 3).^7^ Although no increase in the observed occurrence of ocular harms was observed with robot-assisted surgery, studies with greater sample size and longer follow-up periods are required to conclusively establish the safety of these novel techniques.

## Discussion

Robotic systems have been tested in a wide variety of ophthalmological surgical procedures, ranging from corneal transplantation to subretinal cannulation and infusion. Thus far, RCTs facilitating fair comparisons between robot-assisted and conventional surgery have only been conducted for vitreoretinal procedures such as subretinal tPA infusion or ERM peeling, all using the Preceyes Surgical System.^8^^,^^18^^,^^20^ The Preceyes Surgical System, KU Leuven surgical system, da Vinci Surgical System, and Medineering Robotic Endoscope Guidance System have been trialled in uncontrolled studies. In general, robot-assisted surgery exhibits good efficacy, comparing well to conventional techniques in comparative experiments and with no concerns raised regarding ocular harms. However, despite appearing to improve the efficiency of instrument movements and reducing the physical burden of procedures for surgeons, robot assistance was associated with significant impracticalities, particularly increased procedural duration and perioperative requirements. Part of the increased time of robot-assisted surgery is related to a relative lack of experience, which may be overcome in the future using specialized training modules or virtual-reality surgical training systems such as the Eyesi Surgical Simulator.^26^^,^^27^

In order for robotic assistance to be incorporated into routine surgical workflows, systems must also develop improved integration, ergonomics, and interface with existing surgical set-ups (e.g., surgical tables, patient headrests, operating microscopes, sterile draping systems). This must be achieved without compromising clinical effectiveness or safety. Although current robotic systems have demonstrated safety, integration and effectiveness are areas which require further improvement. Emerging systems demonstrated in animal models and in simulation settings exhibit various innovations that could support clinical use cases. Force sensors can provide feedback to the operator and inform safety algorithms built into the robotic systems.^28^ Remote-control systems could accelerate preparation time by allowing surgeons to scrub-in just once (provided they are not required to convert to manual surgery) and could perhaps facilitate broader geographic coverage of populations without usual access to ophthalmological expertise, without the expense and impracticality of travel for surgeons and patients.^29^ Intraoperative optical coherence tomography (OCT), such as through a sensor integrated into a surgical instrument, may also improve effectiveness and safety.^30^

All trialled systems exhibited a “master–slave” relationship, where the surgeon remains in control of the strategy and techniques of the procedure.^31^ Development of robotic systems with a greater degree of autonomy may help pave the way to automated surgical steps with surgeons remaining in control of selection and supervision of the automated technology.^1^^,^^32^ Novel approaches may draw on training paradigms exemplified by emerging machine learning technology or technological advances in miniaturization.^1^^,^^33^^–^^35^ Such machines may execute subtasks independently (e.g., suturing), generate surgical strategies, and even complete entire procedures without clinician intervention.^1^^,^^31^ However, in the immediate future, research and development are likely to build upon existing robotic systems to extend capability, improve practicality, and safeguard patients as surgical practice evolves. These systems correspond to “Level 0” and “Level 1” surgical robots and are therefore compatible with existing regulatory structures.^1^^,^^36^

The endpoints used in studies included in this review indicate potential advantages of innovative robotic surgical systems in ophthalmology in the future. These focused on technical effectiveness (e.g., surgical success, procedure-specific indicators), safety (e.g., complication rate), and practicality (e.g., procedure duration); successful interventions involving robotic systems should match or exceed conventional surgery in those domains. No studies used patient-reported outcome measures as endpoints, thus failing to capture part of the perspective from the most important stakeholders in surgical innovation. The IDEAL (Idea, Development, Exploration, Assessment, and Long-term monitoring) framework has been adapted to guide innovators toward gold-standard approaches to designing validation studies for robotic surgical systems, and emphasizes incorporation of patients’ perspectives.^37^ Robotic systems have been successfully conceptualized and developed (IDEAL stages 1 and 2a) and have now demonstrated efficacy in a variety of ophthalmic procedures (IDEAL stage 2b). Exploration of potential applications and testing new iterations of robotic systems will continue as researchers aim to optimize for effectiveness, practicality, and safety (IDEAL stage 2b). For clinical validation (IDEAL stage 3), sufficiently powered prospective trials are necessary, with appropriate clinical endpoints used to quantify success in comparisons with manual surgery (or standard of care in situations where manual surgery is infeasible). Validated systems are then required to undergo long-term surveillance to ensure that patients are not adversely affected outside the setting of clinical trials (IDEAL stage 4).^37^

This review was limited by the lack of large RCTs, which precluded conclusions about the clinical effectiveness of robot-assisted surgery relative to conventional manual procedures. Most studies were uncontrolled case series or case reports, successfully proving the feasibility of using robotic systems to complete surgical procedures without providing evidence about whether robot-based interventions should be implemented clinically. All RCTs involved vitreoretinal procedures, implying that other subspecialties are currently less likely to adopt robotic systems.^8^^,^^18^^,^^20^ This lack of comparative trials precluded meta-analysis, as anticipated during design of the review protocol.

Further work is required to develop robotic systems that offer tangible benefits over conventional manual eye surgery. Specific directions for research and development may emphasize facilitation of procedures currently infeasible for human surgeons, or improvements in efficiency to ameliorate the increased procedural duration currently associated with robotic assistance.^38^ To demonstrate benefit, robust RCTs are required to balance the effect of confounding factors between intervention and control arms, minimizing the effect of bias on measured outcomes.^37^ Trials should be sufficiently powered to detect the minimal clinically important difference (MCID) in outcomes of interest, which may include clinical outcomes, such as visual acuity; safety outcomes, such as observed retinotomies; and practicality outcomes, such as duration of surgery. Where the MCID is unclear, such as for microtraumatic events or procedure duration, consensus-seeking initiatives involving relevant stakeholders—especially surgeons and patients—are warranted.

## Conclusions

Robotic systems have demonstrated efficacy in ophthalmic surgery in proof-of-concept studies. These systems remain in relatively early stages of development and currently require extra surgical time to accommodate their adoption. Further surgeon training and integration of these devices are required to optimize the clinical effectiveness and practicality of robot-assisted procedures. With further technological advances in artificial intelligence, imaging, and miniaturization, improved systems will have to undergo pragmatic clinical trials powered to measure effects on clinically relevant outcomes. Clinical adoption will require clearer demonstration of superiority over conventional techniques, perhaps in relation to the performance of technically challenging surgical steps. Future innovation in this young field may enable surgeons to improve the accessibility, effectiveness, and practicality of existing ophthalmic procedures, as well as overcome physiological limits, to introduce new options for surgical management of eye disease.

## Supplementary Material

Supplement 1

Supplement 2
