# Supplementary material for: Robot-Assisted Eye Surgery: A Systematic Review of Effectiveness, Safety, and Practicality in Clinical Settings
Source: Transl Vis Sci Technol. 2024 Jun 25;13(6):20. doi: 10.1167/tvst.13.6.20 (PMC11210629; doi:10.1167/tvst.13.6.20)
Supplement: Supplement 1 [file tvst-13-6-20_s001.docx]

**Supplementary material 1: Literature search undertaken for the systematic review**

**The Cochrane Library**

|  | **Search Terms** | **N** |
| --- | --- | --- |
| **#1** | [mh Ophthalmology] OR [mh “Ophthalmologic Surgical Procedures”] OR [mh “Eye Disease”] OR [mh Eye] OR [mh “Eye Surgery”] OR [mh “Visual Disorder”] OR (eye OR ophthalmolog* OR vision disorders):ti,ab,kw | 32,993 |
| **#2** | [mh “Glaucoma, Angle-Closure”] OR [mh “Glaucoma, Open-Angle”] OR [mh Glaucoma] OR [mh “Glaucoma, Neovascular”] OR [mh “Glaucoma Drainage Implants”] OR [mh “Low Tension Glaucoma”] OR glaucoma:ti,ab,kw | 8,769 |
| **#3** | [mh Retina] OR [mh “Retinal Diseases”] OR [mh “Diabetic Retinopathy”] OR [mh “Epiretinal Membrane”] OR [mh “Retinal Artery Occlusion”] OR [mh “Retinal Degeneration”] OR [mh “Macular Degeneration”] OR [mh “Retinal Detachment”] OR [mh “Retinal Haemorrhage”] OR [mh “Retinal Neovascularization”] OR [mh “Retinal Vasculitis”] OR [mh “Retinal Drusen”] OR [mh “Retinal Vein Occlusion”] OR [mh Retinitis] OR [mh Chorioretinitis] OR [mh “Retinopathy of Prematurity”] OR [mh “Vitreoretinopathy, Proliferative”] OR [mh “Vitreous Detachment”] OR (retina* OR retinopath* OR vitreoretinopath* OR epiretinal membrane* OR macular degenerat* OR maculopath* OR (macula* adj (edema* or oedema*)) OR retinitis OR chorioretinitis OR retinopathy OR vitreoretinal):ti,ab,kw | 18,773 |
| **#4** | [mh Cornea] OR [mh “Corneal Diseases”] OR [mh “Corneal Opacity”] OR cornea*:ti,ab,kw | 11,284 |
| **#5** | [mh “Cataract Extraction”] OR [mh Cataract] OR (cataract OR phacoemulsification):ti,ab,kw | 9,454 |
| **#6** | [mh “Scleral Diseases”] OR [mh “Uveal Diseases”] OR [mh “Choroid Diseases”] OR [mh “Eyelid Diseases”] OR [mh “Iris Diseases”] OR [mh Uveitis] OR [mh Panuveitis] OR [mh “Uveitis, Anterior”] OR [mh “Uveitis, Posterior”] OR [mh “Uveitis, Intermediate”] OR (iridocyclitis OR iritis OR uveitis OR panuveitis):ti,ab,kw | 3,430 |
| **#7** | [mh Strabismus] OR strabismus:ti,ab,kw | 1,453 |
| **#8** | [mh Blepharitis] OR [mh Chalazion] OR [mh Ectropion] OR [mh Entropion] OR [mh Hordeolum] OR (blepharitis OR chalazion OR ectropion OR entropion OR hordeolum):ti,ab,kw | 636 |
| **#9** | [mh “Eye Neoplasms”] OR [mh “Conjunctival Neoplasms”] OR [mh “Eyelid Neoplasms”] OR [mh “Orbital Neoplasms”] OR [mh “Retinal Neoplasms”] OR [mh Retinoblastoma] OR [mh “Uveal Neoplasms”] OR [mh “Choroid Neoplasms”] OR [mh “Iris Neoplasms”] OR (retinoblastoma* OR (conjunctival adj (neoplasm* OR tumo?r* OR malignan* OR cancer*)) OR (iris adj (neoplasm* OR tumo?r* OR malign* OR cancer*)) OR (orbital adj (neoplasm* OR tumo?r* OR malignan* OR cancer*))):ti,ab,kw | 361 |
| **#10** | #1 OR #2 OR #3 OR #4 OR #5 OR #6 OR #7 OR #8 OR #9 | 53,313 |
| **#11** | [mh “Robotic Surgical Procedures”] OR [mh Robotics] OR (robot* OR droid OR bot):ti,ab,kw | 7,369 |
| **#12** | [mh “Surgical Procedures, Operative”] OR [mh “Vitreoretinal Surgery”] OR [mh “Corneal Surgery, Laser”] OR [mh “Operative Time”] OR [mh “Refractive Surgical Procedures”] OR [mh “Laser Therapy”] OR [mh “Minimally Invasive Surgical Procedures”] OR [mh “Eye Enucleation”] OR [mh “Minor Surgical Procedures”] | 167,094 |
| **#13** | (surg* OR transplant* OR incision* OR suture* OR extract*):ti,ab,kw | 387,557 |
| **#14** | #12 OR #13 | 435,730 |
| **#15** | #10 AND #11 AND #14 | 46 |

**Embase**

|  | **Search Terms** | **N** |
| --- | --- | --- |
| **#1** | exp Ophthalmology/ OR exp Ophthalmologic Surgical Procedures/ OR exp Eye Disease/ OR exp Eye/ OR exp Eye Surgery/ OR exp Visual Disorder/ OR (eye OR ophthalmolog* OR vision disorders).mp. | 1,346,439 |
| **#2** | exp Glaucoma, Angle-Closure/ OR exp Glaucoma, Open-Angle/ OR exp Glaucoma/ OR exp Glaucoma, Neovascular/ OR exp Glaucoma Drainage Implants/ OR exp Low Tension Glaucoma/ OR glaucom*.mp. | 114,476 |
| **#3** | exp Retina/ OR exp Retinal Diseases/ OR exp Diabetic Retinopathy/ OR exp Epiretinal Membrane/ OR exp Retinal Artery Occlusion/ OR exp Retinal Degeneration/ OR exp Macular Degeneration/ OR exp Retinal Detachment/ OR exp Retinal Haemorrhage/ OR exp Retinal Neovascularization/ OR exp Retinal Vasculitis/ OR exp Retinal Drusen/ OR exp Retinal Vein Occlusion/ OR exp Retinitis/ OR exp Chorioretinitis/ OR exp Retinopathy of Prematurity/ OR exp Vitreoretinopathy, Proliferative/ OR exp Vitreous Detachment/ OR (retina* OR retinopath* OR vitreoretinopath* OR epiretinal membrane* OR macular degenerat* OR maculopath* OR (macula* adj (edema* or oedema*)) OR retinitis OR chorioretinitis OR retinopathy OR vitreoretinal).mp. | 511,803 |
| **#4** | exp Cornea/ OR exp Corneal Diseases/ OR exp Corneal Opacity/ OR cornea*.mp. | 185,585 |
| **#5** | exp Cataract Extraction/ OR exp Cataract/ OR (cataract OR phacoemulsification).mp. | 111,935 |
| **#6** | exp Scleral Diseases/ OR exp Uveal Diseases/ OR exp Choroid Diseases/ OR exp Eyelid Diseases/ OR exp Iris Diseases/ OR exp Uveitis/ OR exp Panuveitis/ OR exp Uveitis, Anterior/ OR exp Uveitis, Posterior/ OR exp Uveitis, Intermediate/ OR (iridocyclitis OR iritis OR uveitis OR panuveitis).mp. | 157,850 |
| **#7** | exp Strabismus/ OR strabismus.mp. | 32,671 |
| **#8** | exp Blepharitis/ OR exp Chalazion/ OR exp Ectropion/ OR exp Entropion/ OR exp Hordeolum/ OR (blepharitis OR chalazion OR ectropion OR entropion OR hordeolum).mp. | 11,082 |
| **#9** | exp Eye Neoplasms/ OR exp Conjunctival Neoplasms/ OR exp Eyelid Neoplasms/ OR exp Orbital Neoplasms/ OR exp Retinal Neoplasms/ OR exp Retinoblastoma/ OR exp Uveal Neoplasms/ OR exp Choroid Neoplasms/ OR exp Iris Neoplasms/ OR (retinoblastoma* OR (conjunctival adj (neoplasm* OR tumo?r* OR malignan* OR cancer*)) OR (iris adj (neoplasm* OR tumo?r* OR malign* OR cancer*)) OR (orbital adj (neoplasm* OR tumo?r* OR malignan* OR cancer*))).mp. | 84,049 |
| **#10** | #1 OR #2 OR #3 OR #4 OR #5 OR #6 OR #7 OR #8 OR #9 | 1,442,569 |
| **#11** | exp Robotic Surgical Procedures/ OR exp Robotics/ OR (robot* OR droid OR bot).mp. | 123,219 |
| **#12** | exp Surgical Procedures, Operative/ OR exp Vitreoretinal Surgery/ OR exp Corneal Surgery, Laser/ OR exp Operative Time/ OR exp Refractive Surgical Procedures/ OR exp Laser Therapy/ OR exp Minimally Invasive Surgical Procedures/ OR exp Eye Enucleation/ OR exp Minor Surgical Procedures/ | 5,796,770 |
| **#13** | (surg* OR transplant* OR incision* OR suture* OR extract*).mp. | 7,178,428 |
| **#14** | #12 OR #13 | 8,514,417 |
| **#15** | #10 AND #11 AND #14 | 1,246 |

**MEDLINE**

|  | **Search Terms** | **N** |
| --- | --- | --- |
| **#1** | exp Ophthalmology/ OR exp Ophthalmologic Surgical Procedures/ OR exp Eye Disease/ OR exp Eye/ OR exp Eye Surgery/ OR exp Visual Disorder/ OR (eye OR ophthalmolog* OR vision disorders).mp. | 869,448 |
| **#2** | exp Glaucoma, Angle-Closure/ OR exp Glaucoma, Open-Angle/ OR exp Glaucoma/ OR exp Glaucoma, Neovascular/ OR exp Glaucoma Drainage Implants/ OR exp Low Tension Glaucoma/ OR glaucom*.mp. | 82,457 |
| **#3** | exp Retina/ OR exp Retinal Diseases/ OR exp Diabetic Retinopathy/ OR exp Epiretinal Membrane/ OR exp Retinal Artery Occlusion/ OR exp Retinal Degeneration/ OR exp Macular Degeneration/ OR exp Retinal Detachment/ OR exp Retinal Haemorrhage/ OR exp Retinal Neovascularization/ OR exp Retinal Vasculitis/ OR exp Retinal Drusen/ OR exp Retinal Vein Occlusion/ OR exp Retinitis/ OR exp Chorioretinitis/ OR exp Retinopathy of Prematurity/ OR exp Vitreoretinopathy, Proliferative/ OR exp Vitreous Detachment/ OR (retina* OR retinopath* OR vitreoretinopath* OR epiretinal membrane* OR macular degenerat* OR maculopath* OR (macula* adj (edema* or oedema*)) OR retinitis OR chorioretinitis OR retinopathy OR vitreoretinal).mp. | 358,821 |
| **#4** | exp Cornea/ OR exp Corneal Diseases/ OR exp Corneal Opacity/ OR cornea*.mp. | 144,889 |
| **#5** | exp Cataract Extraction/ OR exp Cataract/ OR (cataract OR phacoemulsification).mp. | 78,450 |
| **#6** | exp Scleral Diseases/ OR exp Uveal Diseases/ OR exp Choroid Diseases/ OR exp Eyelid Diseases/ OR exp Iris Diseases/ OR exp Uveitis/ OR exp Panuveitis/ OR exp Uveitis, Anterior/ OR exp Uveitis, Posterior/ OR exp Uveitis, Intermediate/ OR (iridocyclitis OR iritis OR uveitis OR panuveitis).mp. | 95,414 |
| **#7** | exp Strabismus/ OR strabismus.mp. | 22,412 |
| **#8** | exp Blepharitis/ OR exp Chalazion/ OR exp Ectropion/ OR exp Entropion/ OR exp Hordeolum/ OR (blepharitis OR chalazion OR ectropion OR entropion OR hordeolum).mp. | 6,704 |
| **#9** | exp Eye Neoplasms/ OR exp Conjunctival Neoplasms/ OR exp Eyelid Neoplasms/ OR exp Orbital Neoplasms/ OR exp Retinal Neoplasms/ OR exp Retinoblastoma/ OR exp Uveal Neoplasms/ OR exp Choroid Neoplasms/ OR exp Iris Neoplasms/ OR (retinoblastoma* OR (conjunctival adj (neoplasm* OR tumo?r* OR malignan* OR cancer*)) OR (iris adj (neoplasm* OR tumo?r* OR malign* OR cancer*)) OR (orbital adj (neoplasm* OR tumo?r* OR malignan* OR cancer*))).mp. | 60,884 |
| **#10** | #1 OR #2 OR #3 OR #4 OR #5 OR #6 OR #7 OR #8 OR #9 | 989,045 |
| **#11** | exp Robotic Surgical Procedures/ OR exp Robotics/ OR (robot* OR droid OR bot).mp. | 79,516 |
| **#12** | exp Surgical Procedures, Operative/ OR exp Vitreoretinal Surgery/ OR exp Corneal Surgery, Laser/ OR exp Operative Time/ OR exp Refractive Surgical Procedures/ OR exp Laser Therapy/ OR exp Minimally Invasive Surgical Procedures/ OR exp Eye Enucleation/ OR exp Minor Surgical Procedures/ | 3,550,869 |
| **#13** | (surg* OR transplant* OR incision* OR suture* OR extract*).mp. | 5,289,291 |
| **#14** | #12 OR #13 | 6,635,756 |
| **#15** | #10 AND #11 AND #14 | 445 |

**Scopus**

|  | **Search Terms** | **N** |
| --- | --- | --- |
| **#1** | TITLE-ABS-KEY(ophthal* OR ocular OR eye) | 1,106,311 |
| **#2** | TITLE-ABS-KEY(glaucom*) | 117,555 |
| **#3** | TITLE-ABS-KEY(retina* OR retinopath* OR vitreoretinopath* OR *retinitis or "epiretinal membrane" OR macula* OR maculopathy* OR "vitreous detachment") | 487,663 |
| **#4** | TITLE-ABS-KEY(cornea* or kerato* or keratic) | 237,363 |
| **#5** | TITLE-ABS-KEY(cataract* OR phacoemulsification) | 124,654 |
| **#6** | TITLE-ABS-KEY(sclera* OR uvea* OR choroid* OR iris OR iridocyclitis OR iritis OR *uveitis) | 208,478 |
| **#7** | TITLE-ABS-KEY(strabismus) | 34,230 |
| **#8** | TITLE-ABS-KEY(blepharitis OR chalazion OR ectropion OR entropion OR hordeolum OR orbit*) | 613,748 |
| **#9** | TITLE-ABS-KEY(retinoblastoma* OR (conjunctiva* OR iris OR orbit*) AND (neoplasm* OR tumo?r* OR malignan* OR cancer*)) | 57,807 |
| **#10** | #1 OR #2 OR #3 OR #4 OR #5 OR #6 OR #7 OR #8 OR #9 | 2,175,422 |
| **#11** | TITLE-ABS-KEY(robot* OR droid OR bot) | 652,358 |
| **#12** | TITLE-ABS-KEY(surg* OR transplant* OR incision* OR suture* OR extract*) | 7,796,566 |
| **#13** | #10 AND #11 AND #12 | 2,748 |

**Web of Science**

|  | **Search Terms** | **N** |
| --- | --- | --- |
| **#1** | TS=(ophthal* OR ocular OR eye) | 670,639 |
| **#2** | TS=(glaucom*) | 79,246 |
| **#3** | TS=(retina* OR retinopath* OR vitreoretinopath* OR *retinitis or "epiretinal membrane" OR macula* OR maculopathy* OR "vitreous detachment") | 366,657 |
| **#4** | TS=(cornea* or kerato* or keratic) | 153,561 |
| **#5** | TS=(cataract* OR phacoemulsification) | 69,022 |
| **#6** | TS=(sclera* OR uvea* OR choroid* OR iris OR iridocyclitis OR iritis OR *uveitis) | 140,676 |
| **#7** | TS=strabismus | 11,480 |
| **#8** | TS=(blepharitis OR chalazion OR ectropion OR entropion OR hordeolum OR orbit*) | 487,863 |
| **#9** | TS=(retinoblastoma* OR (conjunctiva* OR iris OR orbit*) AND (neoplasm* OR tumo?r* OR malignan* OR cancer*)) | 37,430 |
| **#10** | #1 OR #2 OR #3 OR #4 OR #5 OR #6 OR #7 OR #8 OR #9 | 1,596,781 |
| **#11** | TS=(robot* OR droid OR bot) | 390,374 |
| **#12** | TS=(surg* OR transplant* OR incision* OR suture* OR extract*) | 5,071,272 |
| **#13** | #10 AND #11 AND #12 | 1,442 |
