# Supplementary material for: Robot-Assisted Eye Surgery: A Systematic Review of Effectiveness, Safety, and Practicality in Clinical Settings
Source: Transl Vis Sci Technol. 2024 Jun 25;13(6):20. doi: 10.1167/tvst.13.6.20 (PMC11210629; doi:10.1167/tvst.13.6.20)
Supplement: Supplement 2 [file tvst-13-6-20_s002.docx]

**Supplementary Material 2: Critical appraisal questions used for quality assessment, from Joanna Briggs Institute tools for critical appraisal**

*Case reports*

1. Was the patient’s demographic characteristics clearly described?
2. Was the patient’s history clearly described and presented as a timeline?
3. Was the current clinical condition of the patient on presentation clearly described?
4. Were diagnostic tests or assessment methods and the results clearly described?
5. Was the intervention(s) or treatment procedure(s) clearly described?
6. Was the post-intervention clinical condition clearly described?
7. Were adverse events (harms) or unanticipated events identified and described?
8. Does the case report provide takeaway lessons?

*Case series*

1. Were there clear criteria for inclusion in the case series?
2. Was the condition measured in a standard, reliable way for all participants included in the case series?
3. Were valid methods used for identification of the condition for all participants included in the case series?
4. Did the case series have consecutive inclusion of participants?
5. Did the case series have complete inclusion of participants?
6. Was there clear reporting of the demographics of the participants in the study?
7. Was there clear reporting of clinical information of the participants?
8. Were the outcomes or follow-up results of cases clearly reported?
9. Was there clear reporting of the presenting site(s)/clinic(s) demographic information?
10. Was statistical analysis appropriate?

*Randomised-control trials*

1. Was true randomization used for assignment of participants to treatment groups?
2. Was allocation to treatment groups concealed?
3. Were treatment groups similar at the baseline?
4. Were participants blind to treatment assignment?
5. Were those delivering the treatment blind to treatment assignment?
6. Were treatment groups treated identically other than the intervention of interest?
7. Were outcome assessors blind to treatment assignment?
8. Were outcomes measures in the same way for treatment groups?
9. Were outcomes measured in a reliable way?
10. Was follow-up complete, and if not, were differences between groups in terms of their follow-up adequately described and analyzed?
11. Were participants analyzed in the groups to which they were randomized?
12. Was appropriate statistical analysis used?
13. Was the trial design appropriate and any deviations from the standard RCT design (individual randomization, parallel groups) accounted for in the conduct and analysis of the trial?
